# Cerebellar and/or Brainstem Lesions Indicate Poor Prognosis in Multiple Sclerosis: A Systematic Review

**DOI:** 10.3389/fneur.2022.874388

**Published:** 2022-04-29

**Authors:** Yuyuan Yang, Meng Wang, Lulu Xu, Meixiang Zhong, Yajuan Wang, Moxin Luan, Xingao Li, Xueping Zheng

**Affiliations:** ^1^Department of Geriatric Medicine, The Affiliated Hospital of Qingdao University, Qingdao, China; ^2^Department of Geriatric Medicine, The Qingdao Eighth People's Hospital, Qingdao, China

**Keywords:** multiple sclerosis, cerebellum, brainstem, predictors, disability outcome

## Abstract

Multiple sclerosis is a serious neurological disease that affects millions of people worldwide. Cerebellar and brainstem symptoms are common in the course of multiple sclerosis, but their prognostic value is unclear. This systematic review aimed to determine the relationship between the location of lesions in the cerebellum and/or brainstem and the prognosis in multiple sclerosis. In this systematic review, we searched and comprehensively read articles related to this research topic in Chinese and English electronic databases (PubMed, Embase, Cochrane Library, CNKI, and CBM) using search terms “multiple sclerosis,” “cerebellum,” “brainstem,” “prognosis,” and others. Cerebellar and brainstem clinically isolated syndromes and clinically definite multiple sclerosis were important predictors of transformation (hazard ratio, 2.58; 95% confidence interval, 1.58–4.22). Cerebellar and/or brainstem lesions indicate a poor overall prognosis in multiple sclerosis, but because of inconsistency, more clinical data are needed.

## Introduction

Multiple sclerosis (MS) is a chronic progressive disease of the central nervous system. In patients with MS, approximately 10% to 15% have primary progressive MS, which has a slow disease progression with no remission or recurrence, and 85% to 90% have relapsing-remitting MS (RRMS), which has a marked course of relapse and remission. Of those with RRMS, 75% will progress to secondary progressive MS (SPMS), which has no remission process but has slow progressive exacerbation (1). With the progression of the disease, most patients with MS will develop physical and psychological dysfunction, such as disabilities, cognitive impairment, bladder dysfunction, intestinal dysfunction, anxiety, depression, fatigue, pain, poor sleep quality, and others, which seriously affect the quality of life of patients.

Studies have shown that 81.6% of patients with MS have cerebellar or brainstem clinical manifestations, and 22.5% of the first demyelination events have these two initial clinical manifestations (2). In addition, 10.1% of MS recurrences occur in the cerebellum and 16.6% occur in the brainstem (3). The pons is the most common site of distribution of infratentorial lesions, accounting for 46%, followed by the midbrain, medulla, and cerebellar hemispheres (4).

The cerebellum is responsible for coordination tasks and fine movement. It is believed that the cerebellum plays a key role not only in sensory-motor networks but also in cognitive-behavioral systems. The three predominant cerebellar symptoms (tremor, nystagmus, and scanning speech) were described by the French neurologist Jean-Martin Charcot in 1,877. With the deepening of understanding of MS, cerebellar symptoms are not limited to the three aforementioned symptoms, and gait and truncal ataxia, incoordination of voluntary movements, hypotonia, slurred speech, different types of nystagmus, and ocular dysmetria were also included (5). Studies have shown that cerebellar damage in patients with MS not only leads to motor and cognitive impairment, which hinders daily activities, but is also a marker of poor prognosis (6, 7). Brainstem involvement in MS has different clinical manifestations such as diplopia, facial sensory symptoms, unstable gait, vertigo, facial weakness/hemispasm, oscillopsia, and so forth (8). In addition to these sensory-motor disorders, sleep disorders, restless leg syndrome, and periodic leg movements were also found to be associated with damage to different parts of the brainstem (9). The relationship between brainstem symptoms and the progression of clinically isolated syndromes (CIS) to MS (10–12), which may be 50% to 60%, is also of concern (13). We systematically reviewed studies on cerebellar and brainstem injury and the prognosis of MS, with the aim of identifying patients who may benefit from early, targeted, and positive treatment.

## Methods

**Figure 1:**
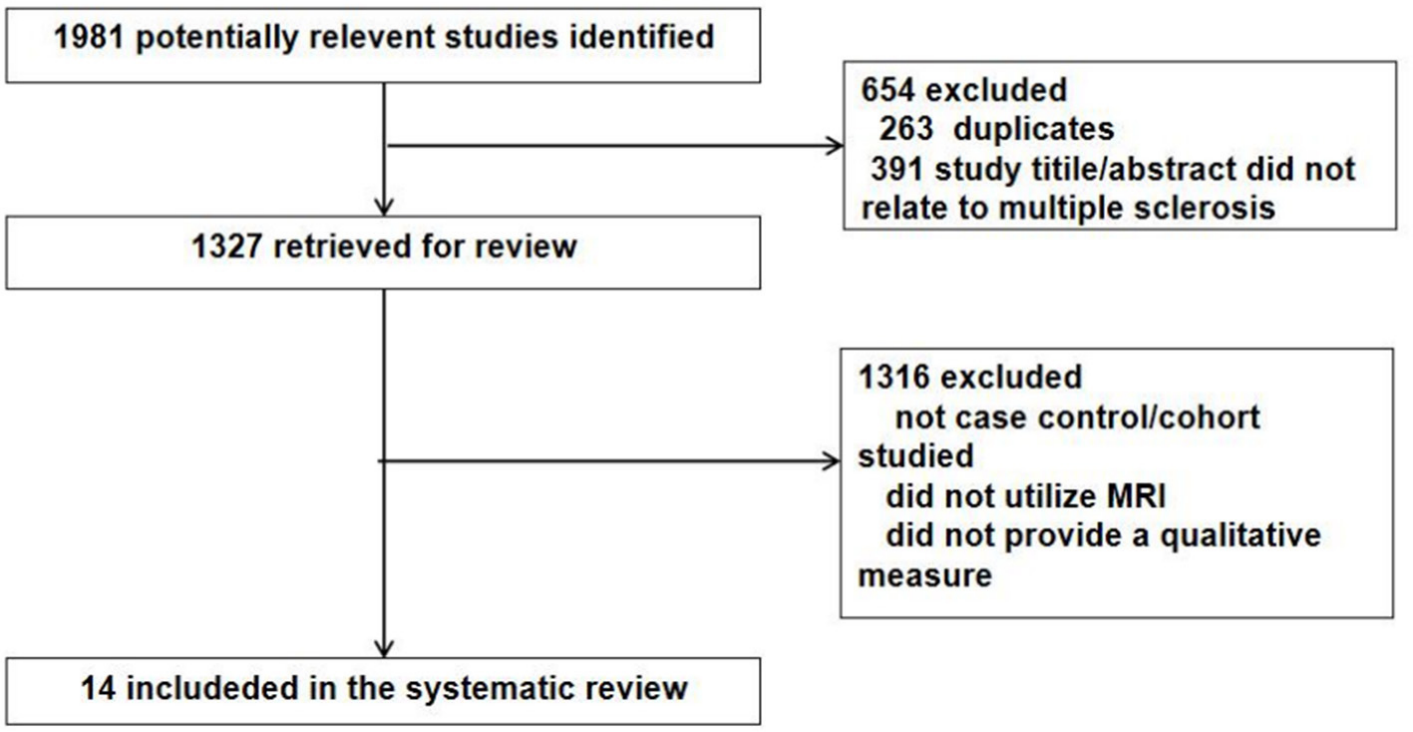
Preferred Reporting Items for Systematic Reviews and Meta-analyses.

Related articles were systematically reviewed according to the Preferred Reporting Items for Systematic Reviews and Meta-analyses (14) guidelines (Figure 1).We searched electronic databases of Chinese and English literature, including PubMed, Embase, the Cochrane Library, CNKI, and CBM. Using a combination of Medical Subject Headings and free text words, a search strategy with both specificity and sensitivity was developed for each database. English keywords included “multiple sclerosis,” “clinical isolation syndrome,” “CIS,” “PPMS” (for primary progressive MS), “SPMS,” “RRMS,” “cerebellum,” “brainstem,” “incidence,” “mortality,” “follow-up study,” “prognosis,” “prediction,” and “course of the disease.” Boolean operators were reasonably used to combine search terms to find relevant research.

The titles and abstracts that may be included in the study were initially screened by two researchers; then, the full text of the preliminary screening literature was read for further in-depth screening. The inclusion criteria were as follows: studies that included patients with MS; the objective of the study is clear and provides prognostic outcome indicators of cerebellar and/or brainstem lesions in MS; articles are in Chinese or English. The exclusion criteria were the following: repeated studies; conference abstracts, case reports, reviews, review abstracts, or quality evaluation of literature research that failed to extract valid information. At the end of the retrieval process, 14 articles (Table 1) were identified and systematically reviewed, including the objectives, methods, results, and limitations of the study. The quality of the included studies was evaluated by two investigators using the Newcastle-Ottawa Scale as an evaluation tool for observational studies on prognostic factors. Stata version 15.0 (Stata Corp., College Station, TX, USA) was used for the statistical analysis of the combined data. The hazard ratio (HR) was first reversed into beta coefficients with standard error, and then the combined HR and 95% confidence interval (CI) were calculated. A comparison of these findings provides a prognostic perspective on the damage to the cerebellum and brainstem in MS and thus aids in understanding the issue.

**Table 1:** Basic characteristics of the included literature.

**References**	**Study design**	**Follow-up (years)**	**Country**	**MS number**	**CIS conversion**	**MS category**	**MS diagnostic criteria**	**Study object**	**MS onset (years)**	**Male/female**	**Conclusion**	**Quality assessment (Newcastle-Ottawa Scale)**
Minneboo et al. (15)	Prospective cohort study	8.7	The Netherlands	42	26 (62%)	CIS	Poser	Brainstem-cerebellum	31.8 (range, 12–52)	25/17	Two or more infratentorial lesions were the best predictors of long-term disability (HR, 6.3)	5
Gaetani et al. (16)	Retrospective cohort study	3.1	Italy	137	116 (84.7%)	CIS	Revised McDonald in 2010	Brainstem-cerebellum	31.4 ± 10.5	102/35	The onset of brainstem-cerebellar symptoms and evidence of a higher baseline MRI lesion load were the strongest independent predictors of early conversion to MS	7
Tintore et al. (17)	Retrospective cohort study	7.7	Spain	246	105 (42.7%)	CIS	Barkhof	Brainstem-cerebellum	30.0 ± 8.5	167/79	The presence of infratentorial lesions increases the risk of disability; brainstem rather than cerebellar lesions may be the cause of adverse events	6
Aurencao et al. (6)	Retrospective cohort study	–	Brazil	122	116 (95.9%)	CIS	Barkhof	Cerebellum	29	33/89	Cerebellar lesions increase the risk of early conversion to MS (OR, 2.4)	5
Banerjee et al. (10)	prospective cohort study	1.5	India	29	5 (17%)	CIS	Revised McDonald in 2010	brainstem	38 (range 20–58)	17/12	CIS with brainstem dysfunction had a higher conversion tendency than other CIS.	4
Çinar et al. (11)	Prospective cohort study	2	Turkey	41	35 (85.4%)	CIS	Poser/McDonald in 2005	Brainstem	–	–	The prognosis of brainstem lesions that transformed into MS was statistically significant	5
Li et al. (12)	Retrospective cohort study	–	HongKong	68	29 (42.6%)	CIS	Barkhof	Brainstem	–	–	CIS presented with brainstem-hemisphere syndrome, and baseline MRI abnormalities were significantly associated with CDMS conversion (*P* < 0.05)	6
Jacome Sanchez and Correa (18)	Retrospective cohort study		Ecuador	117	–	CIS	–	Brainstem	42.2 ± 11.6	31/86	There were no statistically significant differences in the impact of demographic and clinical prognostic factors on disability	5
Phadke et al. (19)	Retrospective cohort study	–	Britain	1055	–	MS	–	Brainstem-cerebellum	–	–	Patients with cerebellar disorders had the shortest survival time at onset, while patients with brainstem injury had the longest survival time.	4
Barzegar et al. (20)	Prospective cohort study	7	Italy	1907	293 (15.4%)	SPMS	–	Cerebellum	–	–	Brainstem dysfunction from RRMS to SPMS is one of the important prognostic factors	4
Nogales-Gaete et al. (21)	Cross-sectional study	–	Chile	314	–	RRMS	–	Brainstem	Range, 12–63	103/210	Brainstem dysfunction at onset was a predictor of treatment failure in the second year	5
Riise et al. (22)	Cross-sectional study	–	Norway	574	–	–	–	Cerebellum	–	–	The presence of pyramidal and cerebellar symptoms at onset predicts a high disability score and rapid transformation to secondary progression	4
Scalfari et al. (23)	Retrospective cohort study		Britain	806	–	SPMS	–	Brainstem-cerebellum	–	–	The time from the onset of progression to EDSS 8 was significantly shorter in patients with a high frequency of early recurrences (≥3 episodes) and with cerebellar and brainstem symptoms	4
Akhtar et al. (24)	Cross-sectional study	–	Kuwait	127	–	POMS	Revised McDonald in 2010	Brainstem-cerebellum	16.0 (range, 6.5–17.9)	36/91	Patients with POMS who have brainstem-cerebellar manifestations are predisposed to SPMS	5

*HR, hazard ratio; MRI, magnetic resonance imaging; OR, odds ratio; CDMS, clinically definite multiple sclerosis; SPMS, secondary progressive multiple sclerosis; EDSS, Extended Disability Status Scale*.

## Results

### Clinically Isolated Syndromes

CIS refers to an single acute or subacute episode of isolated demyelinating events in the central nervous system excluding other diseases (25). The duration of the attack should last at least 24 h, without fever, infection, or clinical features of encephalopathy (26, 27). CIS should not meet the diagnostic criteria of MS in terms of time and space evolution. Studies have found that as many as 80% to 90% of patients with MS had CIS as their first manifestation, and 30% to 70% of patients with CIS progressed into having MS (28). According to the common symptoms and sites of potential involvement, CIS was divided into optic neuritis, myelitis, cerebellar, and brainstem CIS. Cerebellar and brainstem clinical features are often diverse or complex, and they are difficult to locate quickly and accurately. In a retrospective analysis of CIS with brainstem-cerebellar symptoms (assessed within 3 months of onset), the most common symptoms were diplopia (68%), facial sensory symptoms (32%), and gait impairment (31%) (8). Distinguishing brainstem and cerebellar symptoms purely based on clinical presentation is often difficult; thus, they are often discussed together. Studies have found that early drug intervention in CIS can delay the progression to MS (25). Therefore, understanding and identifying the risk factors of clinically definite MS (CDMS) and persistent disease activity (29) can assist in the study of the pathogenesis of demyelination and other diseases and provide a basis for the diagnosis, prognosis, and early intervention of such diseases.

**Figure 2:**
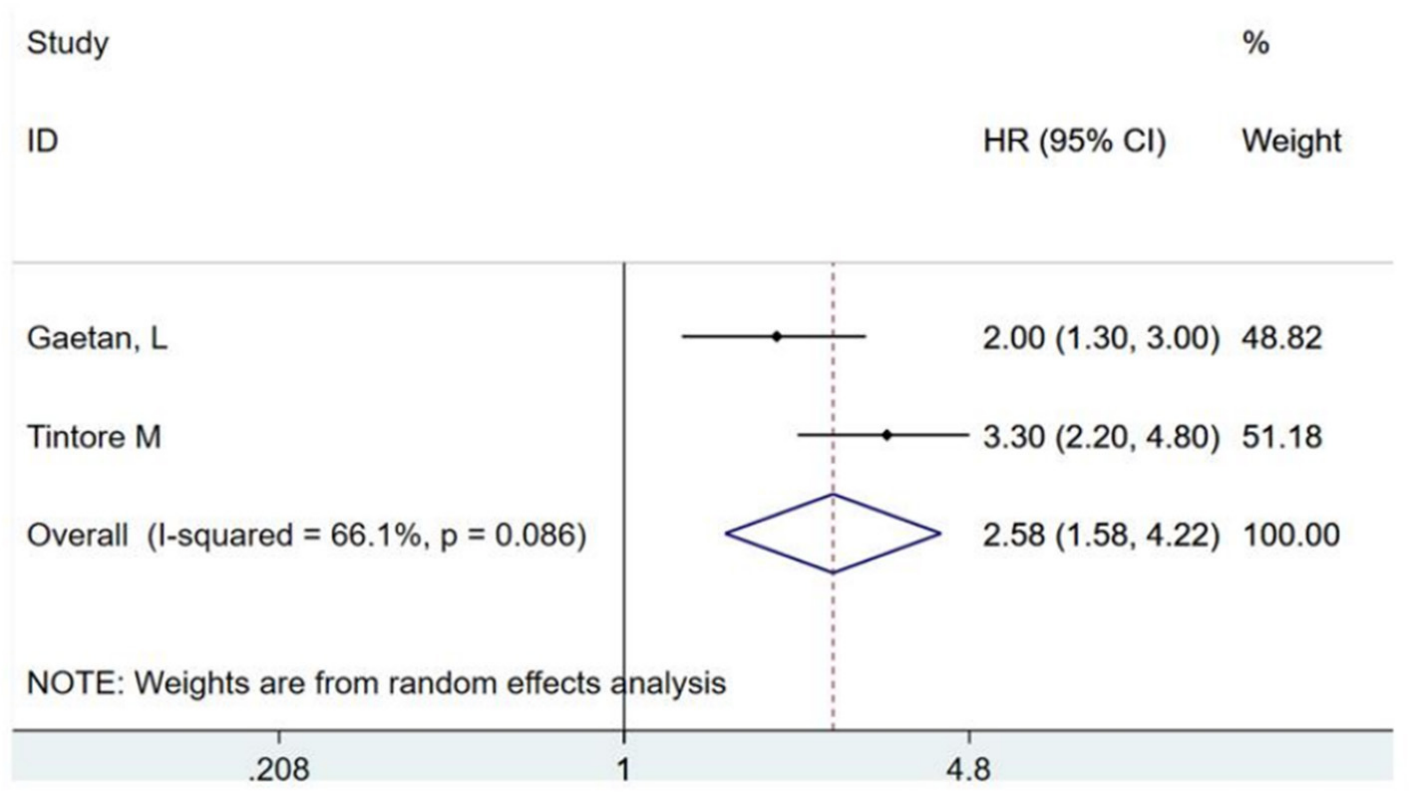
Brainstem and cerebellar lesions predict the risk ratio of conversion of clinically isolated syndromes to clinically definite multiple sclerosis. HR, hazards ratio; CI, confidence interval.

Brainstem-cerebellar CIS is closely related to CDMS and can be used as one of the important predictors of transformation. Two studies (16, 17)have standardized reports and extracted this indicator, with combined HR of 2.58 (95% CI, 1.58–4.22) (as shown in Figure 2). A retrospective multicenter study (16) of 137 patients with a median follow-up duration of 3.1 years found that a clinical onset with brainstem-cerebellar CIS has a higher risk of early conversion to MS (HR, 2.0; 95% CI, 1.3–3.0). In addition, another study indicated that the presence of brainstem-cerebellar lesions not only increases the risk of transformation but also indicates a higher chance of developing a disability (17). A small study of 42 patients with CIS who were followed up for 8.7 years reported that two or more infratentorial diseases were associated with long-term disability (15), which also verified this view.

However, Phadke (19) proposed in 1,990 that patients with primary isolated brainstem lesions had significantly better prognosis (HR, 0.60; 95% CI, 0.38–0.95). Although the study is old, there are still negative outcome reports in recent years, and it is believed that cerebellar or brainstem clinical manifestations at onset have no statistical significance for predicting prognosis (18, 30). These differences can be explained as follows: first, the sample size of each study is small and not sufficiently representative to form persuasive evidence; second, the conversion rate differences may be due to geographic differences in the natural history of MS, length of follow-up, and the use of specific diagnostic criteria. Further prospective and large cohort longitudinal studies are needed to clarify the relationship between the cerebellum/brainstem and CIS conversion to MS.

### Clinically Definite MS

#### Relapsing-Remitting to Secondary Progressive MS

Most patients with MS have a moderate disability (Extended Disability Status Scale [EDSS] ≤ 3) in the relapsing-remitting phase (31, 32), and some patients show little or no disability. After an indefinite period of onset (33, 34), the secondary progressive stage leads to severe disability, inability to live independently, or paralysis and being bedbound. This is due to the accumulation of persistent disability resulting from severe and irreversible nerve damage (with pathological manifestations of axon loss, cortical demyelination, and meningeal inflammatory aggregation) (35). At present, determining the beginning of the secondary progressive course remains difficult; thus, the timing for initiation of effective treatment is unknown. What is clear is that the transition from RRMS to SPMS is a key determinant of prognosis and a primary therapeutic target for the prevention of long-term disability.

The following studies may provide hints for the transformation of RRMS to SPMS. The study of Amato et al. showed that cerebellar manifestations at the onset of MS were associated with a high disability rate and rapid progression to secondary disease (36). Meanwhile, a prospective study of 1,903 patients showed that brainstem dysfunction was one of the important prognostic factors for RRMS conversion to SPMS (20), and brainstem dysfunction at onset was one of the predictors of treatment failure in the second year (21). However, other studies have shown that brainstem symptoms during the onset of MS have no statistical significance in the rate of conversion to SPMS and disability prognosis (22). Scalfari et al. believed that although the type of clinical onset was unrelated to the incubation period from the onset of MS to SPMS, patients with cerebellar and brainstem symptoms at the onset of the disease would have a faster rate of cumulative disability once they reached the secondary progressive stage, and the time from onset to EDSS 8 was significantly shorter (23).

#### Pediatric-Onset MS

Pediatric-onset MS (POMS), defined as MS in persons younger than 18 years, accounts for 2% to 10% of all MS cases and is the most common neuroimmune disorder in children and adolescents (37). The clinical characteristics of patients with POMS are mainly relapse and remission, and its symptoms mostly manifest as brainstem-cerebellar dysfunction (28.6%), pyramidal symptoms (18.4%), and optic neuritis (14.3%). Although POMS have similar a pathogenesis to MS in adults, they have different phenotypic characteristics and disease courses.

Compared with the course of the disease in adults, the early stage of POMS during childhood is characterized by a severe inflammatory process, but the initial recovery of children is better. In addition, POMS has slower disease progression and lower EDSS scores than MS in adults, but the number of relapses is not significantly different (38). In POMS, the cerebellum and brainstem ^(38)^ are the common sites of clinical and radiological involvements (39, 40). Children have a higher volume of infratentorial lesions than adult patients, which means that a higher volume of infratentorial lesions significantly promotes the development of disability due to MS-related cerebellar tissue damage; thus, the association between infratentorial lesions in childhood and disease progression is of particular interest (41).

A Kuwaiti study of prognostic indicators in patients with SPMS who were younger than 18 years highlighted the importance of infratentorial symptoms for prognosis. This finding suggests that brainstem-cerebellar involvement (adjusted HR, 5.71; *P* = 0.010) and onset time of MS (adjusted HR, 1.38; *P* = 0.042) were significantly correlated with the risk of SPMS (24). A retrospective analysis by De Meo et al. showed that baseline evidence of brainstem involvement in patients with POMS was a manifestation of poor prognosis (42). Therefore, patients with brainstem-cerebellar manifestations of POMS are often prone to SPMS, and active treatment is recommended (24).

## Discussion

This systematic review analyzed the relationship between lesion location in the cerebellum and brainstem and disease progression from POMS, CIS, and RRMS to SPMS as well as prognosis. With the continuous development of diagnostic criteria and in-depth understanding of the disease, this time-honored research topic has been refined. Children with MS have a higher incidence of brainstem-cerebellar involvement, which is often a symptom of poor prognosis. In CIS, patients with infratentorial lesions are more likely to be develop CDMS; thus, close attention and timely intervention are needed. In addition to the conversion rate from RRMS to SPMS, infratentorial lesions are also important in disease progression and disability prognosis. One possible explanation is that patients with infratentorial lesions with gait disorders as the main manifestation have higher EDSS scores than other symptoms. Hence, the overall prognosis of MS with cerebellar and brainstem lesions is poor, but more clinical data are needed to support it.

In addition to the typical infratentorial clinical manifestations and signs, imaging examination provides a number of information and plays an important role in the diagnosis and treatment of MS. For example, common cerebellar T2-hyperintense lesions, which were previously believed to have no significant correlation with the extent of lesions and disability (43), have recently been found to be associated with recurrent falls in patients with MS (44). Infratentorial T1-hypointense lesions are associated with disability severity, which is a sign of severe tissue damage and permanent axonal loss (45). Brainstem injury has a good correlation with T2 lesion load (46), but only approximately 60% of the patients with cranial nerve involvement had brainstem lesions identified by magnetic resonance imaging (4), which is called the clinicoradiological paradox. This is a reminder that imaging cannot be viewed in isolation, but must be combined with clinical signs.

With the development of imaging, the advent of diffusion tensor imaging has allowed the use of non-invasive visualization and analysis of white matter (WM) fiber bundles in detecting tissue changes at the micrometer level. In patients with MS, normal WM microstructural changes may precede macro-pathological changes and WM tissue atrophy. The most significant diffusion tensor imaging measurement index for quantitative evaluation of normal WM microstructure changes based on normal distribution is fractional anisotropy (FA) (47). A study in 2016 showed that, during the early MS stage, a normal cerebellum structure in T1- or T2-weighted magnetic resonance imaging showed microstructural changes under high-resolution magnetic resonance imaging and diffusion tensor imaging. Furthermore, cerebellar FA reduction was related to lower EDSS score, disease course, and ratio of cerebellar WM and gray matter volumes, suggesting that the change in the cerebellar microstructure is closely related to disease severity (48). A later study from another perspective showed that the microstructural changes in the cerebellum may be related to the tendency of CIS to convert to MS. If the initial cerebellar FA is lower than FAcrit = 0.352 (where FAcrit is the mean cerebellar FA of patients with early MS), CIS is likely to progress to RRMS within 1 year. This suggests that reduced cerebellar FA in patients with CIS may indicate that the disease is in an active and unstable stage, leading to a shorter transformation time to MS. This series of studies provides us with a more precise perspective in understanding the significance of cerebellar lesions in the early stage of MS (47).

The role of the cerebellum in disability in patients with MS can be explained by the following. First, the cerebellum has important physiological functions. The deep involvement of the cerebellum in motor activities, including working memory, visuospatial function, language, procedural learning, and attention (49), as well as in cognitive (50) and affective processes can seriously affect the quality of life of patients. Second, the cerebellum does not exist in isolation and has extensive connections with other parts of the central nervous system. The cerebellum participates in functional loops with the frontal, superior temporal, limbic, and posterior parietal cortex (48), which is an important factor in the variety of manifestations and adverse outcomes of cerebellum lesions. However, very few studies have focused specifically on brainstem lesions in MS, and there is currently no systematic scientific knowledge.

This systematic review has some limitations. One of the main limitations is the heterogeneity among studies, with differences in patient characteristics, diagnostic criteria, methodology, and outcome reporting. Patient-level data were not available; thus, multivariable predictors of adverse outcomes could not be identified. The meta-analyses and subgroup analyses may be underpowered, and further validation is needed. Most of the studies were cross-sectional and thus provide little insight into the dynamics of the infratentorial involvement in MS. Furthermore, because some of the published studies are older, there are differences in the understanding of MS and infratentorial lesions, which may also influence the outcome.

## Author Contributions

XZ conceptualized the project. YY and MW performed the literature search and screening. LX and MZ evaluated the quality of the articles. YW and ML extracted and analyzed data from the included literature. YY wrote the manuscript with contribution from XZ. All authors read and approved the final manuscript.

## Funding

This work was supported by Clinical +X of The Affiliated Hospital of Qingdao University (QDFY+X202101032).

## Conflict of Interest

The authors declare that the research was conducted in the absence of any commercial or financial relationships that could be construed as a potential conflict of interest.

## Publisher's Note

All claims expressed in this article are solely those of the authors and do not necessarily represent those of their affiliated organizations, or those of the publisher, the editors and the reviewers. Any product that may be evaluated in this article, or claim that may be made by its manufacturer, is not guaranteed or endorsed by the publisher.

## Abbreviations

CIS: clinically isolated syndromes
CDMS: clinically definite multiple sclerosis
EDSS: extended Disability Status Scale
FA: fractional anisotropy
MS: multiple sclerosis
RRMS: relapsing-remitting multiple sclerosis
SPMS: secondary progressive multiple sclerosis
WM: white matter.
